# Transition of Ethiopian highland forests to agriculture-dominated landscapes shifts the soil microbial community composition

**DOI:** 10.1186/s12898-018-0214-8

**Published:** 2018-12-17

**Authors:** Yoseph T. Delelegn, Witoon Purahong, Hans Sandén, Birru Yitaferu, Douglas L. Godbold, Tesfaye Wubet

**Affiliations:** 1 Institute of Forest Ecology (IFE), BOKU – University of Natural Resources and Life Sciences, Peter-Jordan-Straße 82, 1190 Vienna, Austria; 2 Department of Soil Ecology, UFZ-Helmholtz Centre for Environmental Research, Theodor-Lieser-Str. 4, 06120 Halle (Saale), Germany; 3 ARARI–Amhara Regional Agricultural Research Institute, P. O. B. 527, Bahir Dar, Ethiopia; 4 German Centre for Integrative Biodiversity Research (iDiv), Halle-Jena-Leipzig, Leipzig, Germany; 5 Present Address: Department of Community Ecology, UFZ-Helmholtz-Centre for Environmental Research, Halle/Saale, Germany

**Keywords:** Land use change, Soil microbial communities’ composition, ARISA, Soil physicochemical attributes, Ethiopian highlands

## Abstract

**Background:**

Land use changes and related land management practices significantly alter soil physicochemical properties; however, their effects on the soil microbial community structure are still unclear. In this study, we used automated ribosomal intergenic spacer analysis to determine the fungal and bacterial community composition in soils from different land use areas in the Ethiopian highlands. Soil samples were collected from five areas with different land uses, natural forest, eucalyptus plantation, exclosure, grassland and cropland, which had all historically been natural forest.

**Results:**

Our results showed a significant shift in the soil bacterial and fungal community composition in response to land use change. We also identified soil physicochemical factors corresponding to the changes in bacterial and fungal communities. Although most soil attributes, including soil organic carbon, total soil nitrogen, labile P, soil pH and soil aggregate stability, were related to the change in bacterial community composition, the total soil nitrogen and soil organic carbon had the strongest relationships. The change in fungal community composition was correlated with soil nutrients, organic carbon, soil nitrogen and particularly the labile P concentration.

**Conclusions:**

The fungal community composition was likely affected by the alteration of vegetation cover in response to land use change, whereas the bacterial communities were mainly sensitive to changes in soil attributes. The study highlights the higher sensitivity of fungal communities than bacterial communities to land use changes.

**Electronic supplementary material:**

The online version of this article (10.1186/s12898-018-0214-8) contains supplementary material, which is available to authorized users.

## Background

Changes in land use from natural forest to commercial plantation and conventional agricultural landscape is a main driver of biodiversity declines and deterioration of ecosystem services [1–7]. The effects of such transitions from the natural forest to other land uses have been shown to significantly alter soil microbial diversity and community composition, including protozoa, fungi, bacteria and archaea [8–12]. Soil microbes are important regulators of the terrestrial ecosystem, particularly in nutrient-poor ecosystems; soil microbes can enhance plant productivity, plant diversity and species richness, thus maintaining ecosystem processes [13]. The effects of land-use change on the soil microbial community composition are often mediated by changes in the physical, chemical and biological quality of soils [14–16]. However, due to the limited number of studies of this aspect in tropical regions of Africa [17], it is still unclear how land use changes affect the soil microbial community and which factors correspond to such changes. In addition, the high complexity of tropical soil ecosystems, characterized by high levels of species diversity and complex interactions, makes the determination of the prime factors that regulate the microbial community composition difficult [18, 19]. Extensive studies have focused on the link between land use changes and the shift in soil chemical attributes. Some studies are conducted to discern the effect of expansion of commercial plantation at the expense of natural forests, on soil microbes in the tropical rainforest of Southeast Asia and Latin America [5, 20, 21]. However, the effects of land use change on the soil microbial community composition in the tropical regions of Africa has not yet been sufficiently assessed [17]. The soil fungal community composition, for example, are not well considered in any African studies, nor are the effects and impact of human activities on these biological communities is unknown, and thus the need for conservation of belowground diversity may be overlooked [17].

Most of the conversion of natural forests to agricultural land in the northern highlands of Ethiopia has occurred in the last 100 years [22–25]. The historical development and expansion of agriculture and complete dependence on biomass for fuel are the main factors responsible for deforestation in northern Ethiopia [25, 26]. Such natural habitat conversion without consideration of the ecosystem functions became a potential threat to the biodiversity, soil quality, soil microbial communities, belowground processes, and eventually the ecosystem at large [27–29]. Many studies have also revealed that changes in land use result in significant and often long-term impacts on soil nutrient cycles and soil texture [15, 30]. Such change in soil physicochemical parameters plays a role in shifting the composition of the soil fungal and bacterial communities [31–34]. Soil tillage has been reported to decrease soil organic carbon by enhancing rates of organic matter decomposition and leaching and through soil erosion [35]. In the highly populated Ethiopian highlands, removal of plant residues from agricultural lands as sources of fuel and feed for animals is the commonly practiced tradition, which considerably reducing the soil C and inducing soil erosion. Moreover, overgrazing has been shown to have ecological consequences that affect the soil microbial community composition and richness due to its effect on the physical and chemical properties of the soil [36]. In general conversion of natural habitats severely affects the natural vegetation diversity that could play a significant role in regulating soil quality through addition of different quality of organic matter, which will shape the composition of the soil microbial community [37, 38].

The historical transition of natural habitat in Ethiopian highlands provides an interesting insight to investigate and understand the magnitude and direction of change in the composition of soil microbial communities and associated ecosystem functions [22, 23, 39]. In some regional districts of northern Ethiopia, attempts are being made to restore severely degraded lands through area exclosure practices and tree plantations [40, 41], which may also drive a shift in the composition of the soil microbial community. The factors corresponding with the shift in the soil microbial community during restoration are still unexplored. Studies suggest that although there is a high response in soil microbes to land use change, the responses are highly variable from region to region [27]. Thus, understanding the effect of land use changes on the soil attributes provides insights into the complex set of factors that affect soil microbial community composition. In this study, we aimed to investigate how soil bacterial and fungal community composition shift in response to land use change and to identify the soil-related factors that correspond to the bacterial and fungal community composition. To do this, we examined microbial community composition in a natural forest, in cropland and grassland that had been converted from natural forests, and in exclosure and eucalyptus plantations that are presently being used to restore soils of degraded cropland and grasslands in these areas, and buffering the remnant natural forest, respectively. The investigation was carried out within a watershed in the Ethiopian highlands.

## Materials and methods

### Site description

**Fig. 1:**
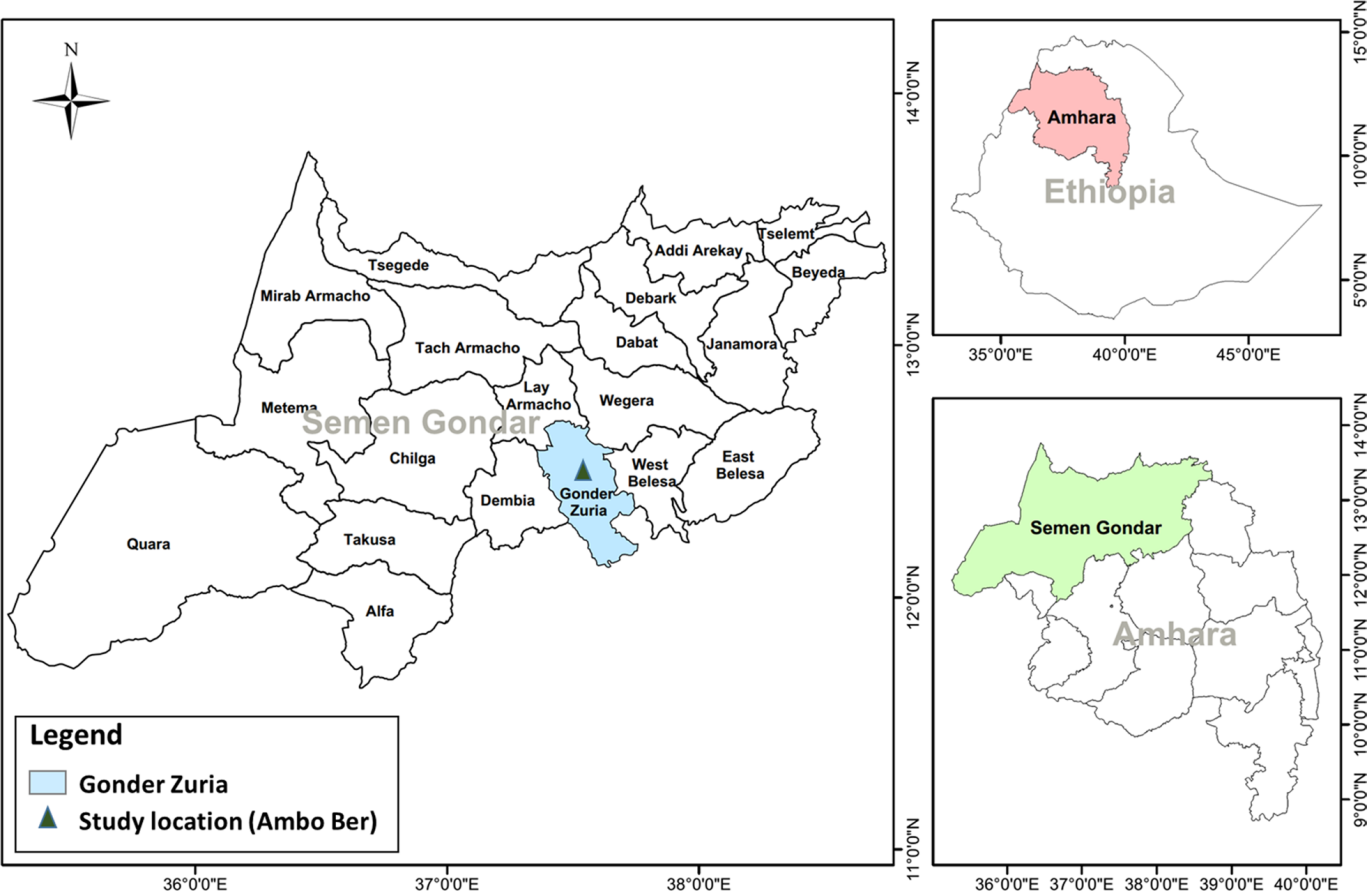
Map of the study area in the Ambo Ber district. The map was produced using ESRI ArcGIS software (version 10.2; http://www.esri.com/software/arcgis/arcgis-fordesktop). The data used for mapping originates from the spatial database of Global Administrative Areas (GADM) (Global Administrative Areas (2016); GADM database of Global Administrative Areas, version 2.8 [online] URL:www.gadm.org)

The study was conducted in Ambo Ber rural district in the North Gondar zone of Amhara Regional State, Northern Ethiopia, located between 12°31′2.87″N and 37°31′24.37″E, approximately 30 km south of the town of Gondar (Fig. 1). Rainfall mainly occurs from June to September. The mean total annual precipitation is 1177 mm. The mean monthly temperature varies from 18 °C in August to 22.5 °C in April. The characteristics of the land uses in the study site are described in detail in a previously published report [42]. Croplands and grasslands in the study area have rapidly been expanding during the last 55 years at the expense of natural forests and shrub lands [22, 23]. By the end of 1970 s, in response to the huge demand for firewood and construction wood from the nearby city and rural towns, the Ethiopian government has established eucalyptus plantation in the project area. In 2007, to support the rehabilitation of the degraded land in Ambo Ber rural district, the University of Natural Resources and Life Sciences, Vienna, has introduced participatory area exclosure practices as appropriate land restoration mechanism.

In this experiment, we examined microbial community composition in the five land uses: (i) a natural forest, covering about 30 ha; (ii) a eucalyptus plantation composed of *Eucalyptus camaldulensis*, covering about 19 ha of land; (iii) a grassland characterized by no pasture management practices and an open grazing system that covers about 3 ha of land; (iv) a cropland characterized by dominant annual crop rotation practices that covers about 4 ha of land; and (v) an exclosure area covering about 2 ha of land [42]. The five land use categories are situated in a watershed at an elevation between 2200 and 2300 m above sea level.

### Soil sampling methods

In mid-February 2015, 7 replicates of soil samples were analyzed per land use type, each replicate consisting of 5 pooled samples, which were taken at least 50–100 m apart along two transect lines (5 land use types × 7 replicates). The soil sampling method was used as described previously [42]. Briefly, two transect lines were taken in each land use type due to the irregular shape of the land uses in the study area. The location of the first sample was randomly selected approximately 50 m from the edge of the land use, and the following samples were taken in 50 or 100 m intervals along the transect lines. The second transect line was set at 50 to 100 m from the first transect, depending on the coverage of the land use area. Each of the seven composite samples was homogenized using a sieve (2 mm) to remove stones, roots, macrofauna, and litter materials.

During soil sample collection and processing, considerable care was taken to avoid contamination of one soil sample by another by using standard laboratory gloves (VWR^®^ nitrile powder-free examination gloves) and cleaning the sieves with 70% ethanol between sieving. The samples were thoroughly homogenized, air-dried and divided into three subsamples; one part was stored at − 20 °C for the analysis of soil microbial community composition, and the second part was stored at + 4 °C for analysis of soil physicochemical properties. The final portion was air dried and kept at room temperature for estimation of arbuscular fungal mycorrhizal spore density. Samples were analyzed at the soil laboratory of Mekelle University, Ethiopia. Soil samples stored at − 20 °C were used for DNA isolation and bacterial and fungal automated ribosomal intergenic spacer analysis (ARISA).

### Analysis of soil properties

Soil chemical properties were analyzed as described in previously published report [42]. Briefly, the soil pH (pH) was determined in 1:2.5 soil suspensions in deionized water using a potentiometric pH meter. Soil organic C (C) and total soil N (N) were analyzed with a LECO CN analyzer (TruSpec^®^ CN, LECO Inc.). The sodium hydroxide and sodium bicarbonate extractable molybdate reactive P (moderately labile P and labile P), being mainly inorganic P, were measured using the modified method described previously [43, 44]. A one g sample of dry soil was used to sequentially extract the phosphorus over 16 h according to the following order: (i) labile P using 30 ml 0.5 M of NaHCO_3_; and (ii) moderately labile P using 30 ml of 0.1 M NaOH [44]. Phosphorus measured by this method is considered mainly orthophosphate, but complex inorganic P or small quantities of organic P hydrolyzed by the molybdate solution could also be included [45].

Soil aggregate stability and arbuscular mycorrhizal fungi spore density were estimated following the methods described previously [42]. Briefly, the soil aggregate stability (SAS) and majorly macro-aggregates (> 0.25 mm diameter) were estimated from 4 g of soil sample using a wet sieving method as described in Delelegn et al. [42]. The SAS was determined as the mass of aggregated soil remaining after wet sieving as a percentage of the total mass of soil without sand materials. The arbuscular mycorrhizal fungi spore density (AMF-SD) was measured using 5 g of soil from wet sieving [46] combined with a sucrose centrifugation method [47, 48] and was estimated under a dissecting microscope as described in INVAM (http://invam.wvu.edu/).

### DNA extraction and bacterial and fungal—ARISA (B- and F-ARISA)

To test the effect of land use change on soil microbial communities, we applied B- and F-ARISA, a culture-independent method, to characterize the microbial community composition across the different land use types. First, 250 mg of soil samples were used to extract DNA using the ZR Soil Microbe DNA MiniPrep Kit (Zymo Research, Irvine, CA, USA), according to the manufacturer’s instructions. NanoDrop measurements with ND-1000 spectrophotometer (Thermo Fisher Scientific, Dreieich, Germany) was primarily conducted to quantify DNA and to check the quality of the isolated genomic DNA. High quality DNA extracts were diluted to ~ 20 ng/µl and the extracts were then stored at − 20 °C until further analysis.

B- and F-ARISA polymerase chain reaction (PCR) amplification was undertaken with the modified method used by Purahong et al. [49, 50] and Yannarell et al. [51]. Briefly, the B-ARISA and F-ARISA technique was employed in two replications under the conditions described by Purahong et al. [52]. Operational taxonomic units (OTUs) derived from ARISA were determined as described in previous studies [52–54]. B-ARISA amplification was conducted by the procedures described by Purahong et al. [50, 52] and Frossard et al. [55]: the PCR mixture (20 μl) contained 1 μl of DNA template (∼ 20 ng of DNA template as determined by the NanoDrop); 10 μM primer 1406f (5′-TGYACACACCGCCCGT-3′) labeled with FAM at the 5′-end and an unlabeled 23Sr primer (5′-GGGTTBCCCCATTCRG-3′); 4 μl of FIREPol 5× Master Mix (Solis BioDyne, Tartu, Estonia); and 13 µl of PCR water to 20 μl. The PCR was carried out with an initial denaturation at 94 °C for 5 min, followed by 30 cycles of 94 °C for 35 s, 55 °C for 45 s, and 72 °C for 2 min, with a final extension at 72 °C for 5 min.

F-ARISA amplification was conducted with the procedures described in Purahong et al. [52]: the PCR mixture (20 μl) contained 1 μl DNA template (∼ 20 ng DNA template as determined by NanoDrop); 10 μM of fungal-specific, plant-excluding primer ITS1F (5′-CTTGGTCATTTAGAGGAAGTAA-3′, [56]) labeled with FAM at the 5′-end and an unlabeled ITS4 primer (5′-TCCTCCGCTTATTGATATGC-3′, [57]; 4 μl FIREPol 5× Master Mix (Solis BioDyne, Tartu, Estonia); and 13 µl of PCR water to 20 μl. PCR was carried out with an initial denaturation at 95 °C for 5 min, followed by 35 cycles of 95 °C for 60 s, 55 °C for 60 s, and 72 °C for 75 s, with a final extension at 72 °C for 7 min. The PCR products were purified using a PCR-Extract Mini Kit (5PRIME, Hamburg, Germany).

As described in Purahong et al. [52], 40 ng (B-ARISA) or 20 ng (F-ARISA) of DNA was mixed with 13.9 µl of deionized Hi-Di formamide (Applied Biosystems, Foster City, CA, USA) and 0.1 μl of internal-size standard Map Maker 1500 ROX (50–1500 bp) (BioVentures, Inc., Murfreesboro, TN, USA). The mixture was denatured for 5 min at 95 °C and chilled immediately on ice for at least 10 min before being further processed using a capillary sequencer (ABI PRISM 3730xl Genetic Analyzer, Applied Biosystems). To optimize the robustness of the ARISA in detecting the change in microbial community composition, the DNA normalization was carried out twice in ARISA (before the initial PCR and before the separation of DNA fragments using capillary electrophoresis).

All peaks of the fragments between 200 and 1500 bp that were present in two analytical PCR replicates were used for further analyses [19, 52]. The two independent PCR replicates were highly correlated. OTU binning was carried out using an interactive custom binning script in R version 2.14.1 [58]. The relative fluorescent intensity (RFI) was determined following the normalization of the total peak areas to 1. All background noises with RFI values lower than 0.09% were cleared. A strategy involving a binning size of 2 bp was applied to the B- and F-ARISA data, and the binning frame that gave the highest pairwise similarity among samples was used for further statistical analyses.

### Statistical analysis

The effects of land use and soil physicochemical properties on the bacterial and fungal community composition were evaluated using PAST [59] and R version 3.2.2 [60], using “vegan” package [61]. The bacterial and fungal OTU richness and Shannon diversity were calculated using the PAST function “diversity indices”. The differences in bacterial and fungal OTU richness and diversity among the five land uses were analyzed for differences among means (*P *< 0.05) by performing one-way analysis of variance (ANOVA) using the PAST program. All data sets were tested for normality and the equality of group variances using a JB test and the Levene statistic. One-way analysis of similarity (ANOSIM) was employed by taking the land use as the main factor to investigate the shifts in the bacterial and fungal community composition across the land uses. ANOSIM was carried out based on the Bray-Curtis distance measure using abundance data to test for significant differences in bacterial and fungal community composition among different land uses. The statistical significances of the differences in both bacterial and fungal community compositions were carried out based on 999 permutations and Bonferroni-corrected *P* values were applied. ANOSIM produces a sample statistic (*R*) that is supposed to vary between the test groups ranging from − 1 to 1 (*R* = 0, no separation; *R* = 0.30 − 0.75, there is separation but with some degree of overlapping; *R* = 1, complete separation) [49]. The ANOSIM produces an overall similarity measure and “Pairwise Tests” of bacterial and fungal OTUs compositions between the land uses.

Non-metric multidimensional scaling (NMDS) was performed to visualize the separation of the bacterial and fungal community compositions between each land use using R. Goodness-of-fit statistics (*R*^2^) for environmental factors fitted to the NMDS ordinations of bacterial and fungal communities were calculated using the “envfit” function in the vegan module in R, with *P* values based on 999 permutations. The goodness-of-fit statistics (*R*^*2*^) provide information about which environmental variables correspond with bacterial and fungal OTU compositions among the land uses. Autocorrelations among soil factors were investigated using Spearman’s rank correlation [62]. Land use-averaged rarefaction curves for bacteria and fungi were generated using ‘sample rarefaction’ in PAST based on seven replicates.

## Results

### Bacterial and fungal OTUs across different land uses

Overall, 307 bacterial and 314 fungal OTUs were detected. Distributions of bacterial and fungal OTUs across different land uses are shown in Table 1. Bacterial OTU richness and diversity were not significantly different among different land uses. In contrast, fungal OTU richness and diversity significantly varied among land uses systems. Fungal OTU richness in soils of grassland samples (54) was significantly lower than OTU richness detected in soils of the eucalyptus plantation (87) and of natural forest (77). Fungal OTU diversity measured in soils of the grassland (2.3) was significantly lower than OTU fungal diversity detected in the natural forest (3.2) and exclosure (3.0) (Table 1). Land use-averaged rarefaction curves for bacteria and fungi are shown in the online Additional file 1: Figure S1.

**Table 1:** Bacterial and fungal OTU richness and Shannon diversity in different land uses

Land use types	Soil microbial community richness		Soil microbial community diversity	
	Bacterial community (Mean ± SE)	Fungal community (Mean ± SE)	Bacterial community (Mean ± SE)	Fungal community (Mean ± SE)
Cropland	87.29 ± 7.08^a^	68.29 ± 6.13^ab^	3.80 ± 0.10^a^	2.81 ± 0.13^ab^
Grassland	75.29 ± 8.05^a^	54.29 ± 7.57^a^	3.75 ± 0.13^a^	2.29 ± 0.18^a^
Exclosure	67.29 ± 6.18^a^	60.71 ± 8.04^ab^	3.58 ± 0.11^a^	2.98 ± 0.12^b^
Eucalyptus plantation	75.00 ± 6.47^a^	86.71 ± 5.75^b^	3.76 ± 0.09^a^	2.78 ± 0.13^ab^
Natural forest	71.86 ± 4.23^a^	76.71 ± 7.84a^b^	3.73 ± 0.05^a^	3.18 ± 0.09^b^

Different letters indicate significant differences (*P* < 0.05)

### Effect of land use changes on bacterial and fungal community composition

**Fig. 2:**
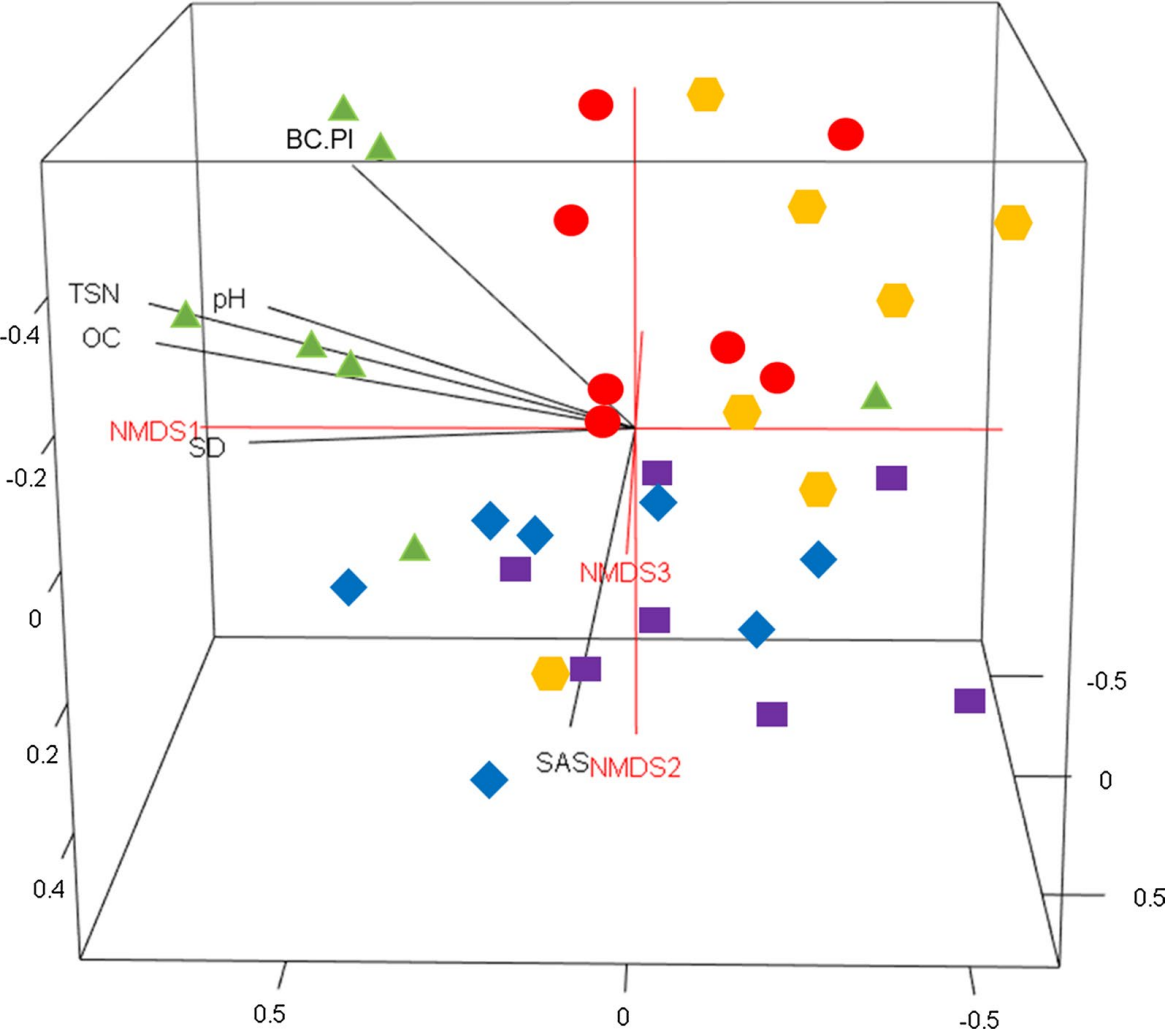
Three-dimensional non-metric dimensional scaling (3D-NMDS) showing the bacterial community composition distributed across five different land uses (  = natural forest,  = Eucalyptus plantation,  = exclosure,  = grassland and  = cropland). SAS: soil aggregate stability; BC. PI: bicarbonate-reactive P; OH. PI: hydroxide-reactive P; OC: organic carbon; TSN: total soil nitrogen; SD: arbuscular mycorrhizal fungal spore density

**Fig. 3:**
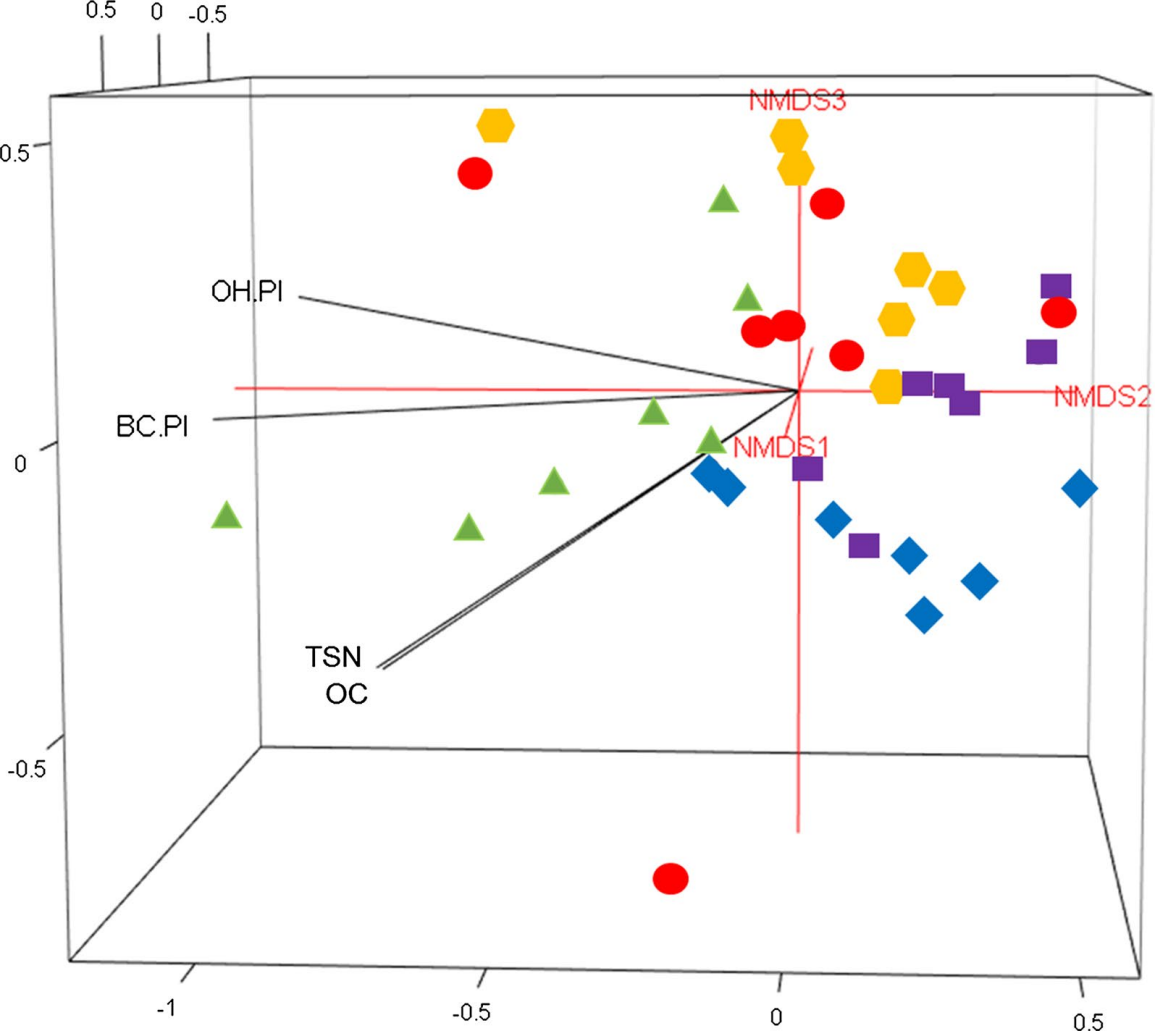
Three-dimensional non-metric dimensional scaling (3D-NMDS) showing the fungal community composition distributed across five different land uses (  = natural forest,  = Eucalyptus plantation,  = exclosure,  = grassland and  = cropland). SAS: soil aggregate stability; BC. PI: bicarbonate-reactive P; OH. PI: hydroxide-reactive P; OC: organic carbon; TSN: total soil nitrogen; SD: arbuscular mycorrhizal fungal spore density

The overall soil microbial community composition was significantly influenced by the land use change (bacterial community: ANOSIM = 0.31, *P* = 0.001; fungal community: ANOSIM = 0.46, *P* = 0.001) (Tables 2 and 3). The ANOSIM for bacterial community indicates more overlapping of the bacterial community compositions across the land uses compared to the fungal community compositions among the land uses (Tables 2 and 3, Figs. 2 and 3). The pairwise ANOSIM for bacterial community composition showed significant differences between natural forest and all other land uses (*P* < 0.05) except for the eucalyptus plantation (*P* = 0.13) (Table 2). The exclosure was also significantly separated from the grassland (Table 2). The pairwise ANOSIM for fungal community composition indicated significant differences in most cases (*P* < 0.05) except between the exclosure and cropland and the grassland and cropland (*P* = 0.36 − 0.99) (Table 3).

**Table 2:** Analysis of similarity (ANOSIM) based on Bray-Curtis and Manhattan distance measures (identical results in all cases) using abundance data comparing bacterial community composition across different land uses

Changes with land use Categories (LU1—LU2)	N	*R*	*P*	OTUs detected (Total OTUs)	Bacterial OTU (%)		
					LU1	LU2	Shared
Natural forest—eucalyptus	14	0.32	0.13	190 Vs. 188 (240)	52 (22)	50 (21)	138 (57)
Natural forest—exclosure	14	*0.41*	*0.01*	190 Vs. 177 (232)	55 (24)	42 (18)	135 (58)
Natural forest—grassland	14	*0.51*	*0.04*	190 Vs. 191 (241)	50 (21)	51 (21)	140 (58)
Natural forest—cropland	14	*0.47*	*0.04*	190 Vs. 206 (259)	53 (20)	69 (27)	137 (53)
Eucalyptus—exclosure	14	0.11	0.99	188 Vs. 177 (227)	51 (23)	39 (17)	137 (60)
Eucalyptus—grassland	14	0.21	0.24	188 Vs. 191 (236)	46 (20)	48 (20)	142 (60)
Eucalyptus—cropland	14	0.23	0.20	188 Vs. 206 (253)	49 (19)	66 (26)	138 (55)
Exclosure—grassland	14	*0.35*	*0.01*	177 Vs. 191 (233)	41 (18)	57 (24)	135 (58)
Exclosure—cropland	14	0.31	0.09	177 Vs. 206 (246)	42 (17)	70 (28)	134 (55)
Grassland—cropland	14	0.16	0.68	191 Vs. 206 (252)	49 (19)	62 (25)	141 (56)

Percentage of OTUs detected in Land Use 1 (LU1), Land Use 2 (LU2) and shared OTUs between LU1 and LU2 are shown for pairwise comparisons. Significant factors (*P* < 0.05) are indicated in italic

*R* degree of separation between test groups ranging from − 1 to 1; *R* 0, not different; *R*  1, completely different (i.e., where the *R*-value between 0–0.299 “no separation/overlapping”; 0.300–0.749 “different but with some overlapping”; and > 0.750 “well separated”); N = population size. *P* values were based on 999 permutations (significant values with Bonferroni correction (*P* < 0.05) are given in italic

**Table 3:** Analysis of similarity (ANOSIM) based on Bray-Curtis and Manhattan distance measures (identical results in all cases) using abundance data comparing fungal community composition across different land uses

Changes with land use Categories (LU1—LU2)	N	*R*	*P*	OTUs detected (Total OTUs)	Fungal OTU (%)		
					LU1	LU2	Shared
Natural forest—eucalyptus	14	*0.70*	*0.02*	203 Vs. 192 (243)	51 (21)	40 (16)	152 (63)
Natural forest—exclosure	14	*0.51*	*0.03*	203 Vs. 177 (231)	54 (23)	29 (13)	148 (64)
Natural forest—grassland	14	*0.67*	*0.02*	203 Vs. 161 (227)	66 (29)	24 (11)	137 (60)
Natural forest—cropland	14	*0.45*	*0.02*	203 Vs. 178 (231)	53 (23)	28 (12)	150 (65)
Eucalyptus—exclosure	14	*0.66*	*0.01*	192 Vs. 177 (230)	52 (23)	37 (16)	141 (61)
Eucalyptus—grassland	14	*0.61*	*0.01*	192 Vs. 161 (223)	60 (27)	29 (13)	134 (60)
Eucalyptus—cropland	14	*0.48*	*0.02*	192 Vs. 178 (228)	50 (22)	36 (16)	142 (62)
Exclosure—grassland	14	*0.35*	*0.02*	177 Vs. 161 (211)	51 (24)	34 (16)	126 (60)
Exclosure—cropland	14	0.16	0.36	177 Vs. 178 (220)	42 (19)	43 (20)	135 (61)
Grassland—cropland	14	0.01	0.99	161 Vs. 178 (212)	34 (16)	52 (25)	126 (59)

Percent of OTUs detected in Land Use 1 (LU1), Land Use 2 (LU2) and shared OTUs between LU1 and LU2 are shown for pairwise comparisons. Significant factors (*P* < 0.05) are indicated in italic

*R* degree of separation between test groups ranging from − 1 to 1; *R* 0, not different; *R* 1, completely different (i.e., where the *R*-value between 0–0.299 “no separation/overlapping”; 0.300–0.749 “different but with some overlapping”; and > 0.750 “well separated”); N = population size. *P* values were based on 999 permutations (significant values with Bonferroni correction (*P* < 0.05), are given in bold

### Factors corresponding with bacterial and fungal community composition

Soil physicochemical properties and arbuscular mycorrhizal fungi spore density (AMF-SD) were significantly affected by land use type (Table 4). Interestingly, Sahner et al. [63], have found an increased AM spore abundance in highly intensive land use systems in comparison to secondary rainforests suggesting that fungi produce spores as resting structures to survive unfavorable conditions. Our findings indicated variability of the soil nutrient concentration (C, N, labile P, and moderately labile P), soil physical attribute (SAS) and AMF-SD following land use changes (Table 4). The soil pH of the eucalyptus plantation was significantly lower than in the exclosure and natural forest. This result also showed that the content of C, N and labile P in the natural forest soil was significantly higher than in the cropland and grassland. For example, C and N were four times higher in the natural forest than in the cropland, and labile P was three times higher in the same comparison (Table 4).

**Table 4:** Soil biological and physicochemical attributes (Mean ± SE) across the five land uses

Land use	AMF-SD	SAS	Soil pH	SOC	TSN	NaHCO3 Mo-P	NaOH Mo-P
Cropland	74.46 ± 9.17^bc^	42.52 ± 3.42^a^	6.61 ± 0.06^ab^	1.96 ± 0.21^a^	0.16 ± 0.01^a^	17.84 ± 2.66^b^	121.98 ± 26.74^b^
Grassland	37.8 ± 8.17^a^	64.37 ± 4.70^b^	6.74 ± 0.04^ab^	3.04 ± 0.34^b^	0.25 ± 0.03^b^	14.16 ± 3.38^b^	82.21 ± 25.20^ab^
Exclosure	85.63 ± 6.31^bc^	70.35 ± 5.22^bc^	6.97 ± 0.12^b^	3.87 ± 0.39^bc^	0.31 ± 0.02^bc^	4.57 ± 0.59^a^	25.84 ± 2.30^a^
Eucalyptus	74.17 ± 5.42^b^	83.82 ± 1.25^c^	6.38 ± 0.13^a^	4.35 ± 0.61^bc^	0.27 ± 0.03^b^	15.20 ± 4.54^ab^	44.64 ± 3.24^ab^
Natural forest	123.86 ± 20.76^c^	62.99 ± 6.70^b^	6.93 ± 0.11^b^	8.22 ± 1.59^c^	0.65 ± 0.13^c^	58.46 ± 12.01^c^	127.48 ± 14.83^b^

Different letters indicate significant differences (*P* < 0.05)

AMF-SD: arbuscular mycorrhizal fungi spore density; SAS: soil aggregate stability; C: soil organic carbon; N: total soil nitrogen; Labile P: molybdate-reactive bicarbonate-extractable P; moderately labile P: molybdate reactive hydroxide-extractable P

The results showed a significant correspondence of the bacterial community composition with the AMF-SD and most of the soil physicochemical properties including SAS, C, N, pH and labile P. Among the soil attributes, C and N showed strong correlations with bacterial community composition (*R*^2^ = 0.64 and *R*^2^ = 0.66, respectively). However, no statistical correspondence was detected between the bacterial community composition and moderately labile P (*P* = 0.13) (Table 5). The fungal community composition was significantly correlated with soil nutrients (C, N, labile P and moderately labile P). In contrast to the bacterial community composition, the SAS, pH and AMF-SD did not show significant correlations with the fungal community composition (*P* > 0.05) (Table 5). Among the soil nutrients, the labile P was highly correlated with the fungal community composition (*R*^2^ = 0.49, *P* = 0.004).

**Table 5:** Goodness-of-fit statistics (*R*^*2*^) of environmental variables fitted to the nonmetric multidimensional scaling (NMDS) ordination of bacterial and fungal communities

Soil attributes	Bacterial communities		Fungal communities	
	*R* ^*2*^	*P* value	*R* ^*2*^	*P* value
AMF spore density (AMF-SD)	*0.412*	0.002**	0.128	0.299
Soil aggregate stability (SAS)	*0.270*	0.038*	0.064	0.642
Soil pH (pH)	*0.287*	0.029*	0.079	0.537
Molybdate-reactive bicarbonate-extractable P (Labile P)	*0.414*	0.003**	*0.486*	0.004**
Molybdate-reactive hydroxide-extractable P (moderately labile P)	0.192	0.127	*0.279*	0.042*
Soil organic carbon (C)	*0.644*	0.001***	*0.393*	0.010**
Total soil nitrogen (N)	*0.660*	0.001***	*0.408*	0.008**

Significant factors (*P* < 0.05) are indicated in italic: *P < 0.05, ** *P* < 0.01, ***P < 0.001

## Discussion

This study clearly demonstrated that a distinctive microbial community was harbored in the natural forest, and the composition of this community was clearly different from all other land uses (Tables 2 and 3; Figs. 2 and 3). These findings are concomitant with a number of studies [20, 52, 64, 65] in which the authors described the significant relationships between soil microbial community composition and biotic and abiotic environmental conditions. Land use change affects major soil physical and chemical attributes [32, 66–68]. The historical transition processes from the natural forest to agricultural land in the tropics has been suggested to induce soil erosion that consequently leads to poorer soil fertility and texture [31, 32, 69]. Our results clearly differentiated the natural forest from the other land uses, particularly from croplands and grasslands. In the topsoil of the natural forest, higher C and N concentrations were observed (Table 4). In contrast, the high concentration of moderately labile P measured in cropland compared to forest is likely due to P addition from fertilizer [70]. The land use change can be an indirect driver for shaping the soil microbial community composition by altering the soil biophysical and chemical attributes [15, 64, 71].

### General overview of the effects of land uses change on microbial richness, diversity and community composition

OTUs derived from ARISA may not be equivalent to species and may differ from those OTU definitions relying on sequences. However, they do provide a basis for richness estimates, and they allow highly consistent measurements of community composition through space and time. In this study, compared to bacteria, fungal richness and diversity appeared to be more affected by the land use change (Table 1). Different responses of the two groups of soil microbial communities to land use changes may be due to their distinct functions in the soil ecosystems and the likelihood of having distinct mineralization processes depending on the nature of organic carbon substrate [72, 73]. The distinct differences in the physiology and ecology of bacterial and fungal communities suggest that the distribution and abundance of each microbial group would be controlled by separate soil biophysical and chemical attributes that vary among the types of land use [74]. Thus, the shifts of the organic carbon and nutrient pools driven by the land uses changes (e.g., vegetation changes) could cause the shifting of the composition of the two microbial communities in different ways.

We have shown that the shift in bacterial community composition (Table 2) was more obvious than the changes in bacterial OTU richness and diversity (Table 1). This may be related to the gain, loss and community rearrangement processes that normally occur in human-disturbed ecosystems [49, 75]. Some bacterial OTUs are lost due to changes from natural forest to other land use types; however, some bacterial OTUs will be gained. If the bacterial community is simply rearranged, meaning that the OTU gain and loss are equal, then we could only detect changes in the community composition. The fungal OTU richness and diversity were found to be more sensitive to land use changes than bacterial OTUs (Table 1). The major reason for the sensitivity of fungi to land use change may be due to the presence of symbiotic associations (mycorrhizal fungi) with vegetation and soil disturbance [76]. Thus, the discrepancy in fungal OTU diversity detected in the grassland was likely to be linked with the limited vegetation diversity in the grassland (dominated by highland perennial grasses) compared to the natural forest and the exclosure, which had the highest plant species diversity.

ARISA-based microbial communities’ composition assessment allows fast, high throughput and low cost identification of microbial OTUs (phylotypes) [77, 78]. Nevertheless, to understand the specific fungal functional groups associated with vegetation diversity, application of DNA-based methods such as Next Generation Sequencing (NGS) is necessary. This method allows assigning taxonomic and functional guilds of the fungal communities and identifying the main players, e.g., the fungal OTUs that exhibit shifts in diversity and community composition in response to the change in vegetation type [79, 80].

### Shift in bacterial community composition

The bacterial community composition of the natural forest was different from the other land uses types except the eucalyptus stand, and the drivers were mainly the autocorrelated C, N and pH. The correspondence we found between the soil pH and bacterial community composition is supported by the findings of a number of studies [10, 15, 71, 81]. The effect of soil pH in shaping the bacterial community composition is described by its effect on the growth and proliferation of some bacterial communities, where bacterial physiological attributes exhibit optimal growth within narrow pH ranges (e.g., *Actinobacteria, Bacteroidetes* and *Acidobacteria*) [15, 82, 83]. In many studies [81–83], soil pH has been demonstrated to be the best predictor of the shift in bacterial community composition. However, we found that soil C and N (*R*^*2*^ = 0.66, *P* < 0.001; *R*^*2*^ = 0.66, *P* < 0.001, respectively) were comparatively stronger predictors of the shifts in bacterial community composition (Table 5). It has also been questioned whether pH itself is the factor that directly shapes the bacterial community composition, as pH is highly correlated with a range of soil parameters [83]. C and N are important elements for microbial growth and survival; this reinforces the strongly significant correlation of C and N with the bacterial community composition [15, 52]. Despite the differences in soil pH, C and N between the natural forest and eucalyptus plantation, the lack of a difference in the bacterial composition between these land uses may result from the recalcitrant litter accumulation in the topsoil layer in the woody land uses compared to the grassland and the cropland [84]. The significant difference in the bacterial community observed between the exclosure and grassland (Table 2) seems not to be only due to the soil C and N content but also due to the higher SAS and lower labile P in the exclosure than in the grassland (Fig. 2). The association of specific bacteria to specific plant species or to local soil conditions created below and/or in the vicinity of the roots of different plants will affect the microbial composition [84, 85].

Interestingly, we found that AMF-SD corresponds with bacterial community composition (Table 5). This may be related to close relationships found between AMF and bacteria [86, 87]. The review conducted by Frey-Klett et al. [88] illustrated the mechanism of how the mycorrhiza-helper bacteria promote the establishment of symbiosis by stimulating mycelial extension, increasing root–fungus contacts and colonization, and reducing the impact of adverse environmental conditions on the mycelium of the mycorrhizal fungi. Several studies have also examined the bacterial strains isolated from the spores of the *Glomus* species. These studies reported the stimulating effect of bacteria on spore germination and AM fungal formation [89, 90] through the production of growth factors that detoxify the antagonistic substances, or through the inhibition of competitors and antagonists [88]. In general, those studies reinforced the close association of AMF and bacterial communities that we observed in this study (Table 5). Our finding shows that the soil attributes can explain the main differences found in bacterial community composition between the different land uses, but the current vegetation may also influence the composition.

### Shift in fungal community composition

Our results indicate that the fungal community composition (Table 3) in Ambo Ber is more sensitive to land use changes than the bacterial community composition (Table 2). Similar to bacteria, the fungal community composition was shown to be correlated with soil C and N, which separates the natural forest from the other land uses (Table 5; Fig. 3). However, the correspondence was weaker than that observed with the bacterial community composition (Table 5). Moreover, we found no significant correspondence with soil pH, SAS and AMF-SD in the fungal community, in contrast to the bacterial community (Table 5; Fig. 3). Soil pH is often a predictive factor for the microbial community composition, but pure culture studies show that fungi generally exhibit a wider pH range for optimal growth than the bacterial communities, which makes the correspondence between soil pH and fungi weaker [83]. Lauber et al. [15], found a similar pattern, where they observed no significant correspondence between the fungal communities and soil pH while there was significant correspondence with the bacterial community composition. Surprisingly, we found no correspondence between SAS and fungal community composition (Table 5). Some studies have found a significant role of the fungal community in the formation of macro-aggregates of soils via physical and biochemical factors including mycelia exudates [91, 92].

Fungal community composition was more strongly correlated with the extractable P, including the labile P and moderately labile P, than bacteria, which were only weakly correlated with labile P. Soil P is often a limiting factor in the arid tropical soil, particularly in sub-Saharan Africa, due to rapid fixation of available P into less soluble P [93]. In this poor soil environment, the role of fungal communities in mineralization of tightly bound P to extractable P is significant [94].

The natural forest and the eucalyptus plantation also differed strongly in fungal community composition, which was not the case for bacteria. In addition to differences in soil C, N and P, the discrepancy may be due to the relatively rich vegetation diversity of the natural forest compared to the eucalyptus forest, where *Eucalyptus camaldulensis* is the dominant species. Dominant tree species are known to significantly influence soil fungal community composition [95]. According to Sun et al. [95], the fungal species richness and community structure can be significantly influenced by the interaction effect between soil and tree species. Tree species may influence the fungal community structure by changing the chemical composition of litter and root exudates and by creating a spatially heterogeneous environment [96]. The study of Sun et al. [84] also described how the forest ecosystems shape the soil microbial community composition due to the addition of the leaf and woody litter to the forest floor. The various types of litter are characterized by different quality of nutrients and by the recalcitrance of the material. In other words, the type of land use (i.e., characterized by the type of vegetation and land management practices) structures the microbial community by dictating the competitive ability of microbial communities to degrade the available organic matter types [84, 85]. Different dominant tree species may also explain the difference in soil fungal community composition between the natural forest, the eucalyptus plantation and the exclosure. In general, the strong separation shown between the relatively less disturbed woody forests (the natural forest and the eucalyptus plantation) and the highly disturbed soil in cropland and grassland could be due to the differences in the accumulation of woody material and the reduction of vegetation biomass and continual biomass removal due to overgrazing [67]. The vegetation changes and disturbances have separated both cropland and grassland from the forested landscapes, while there was very strong overlapping of fungal OTUs between cropland and grassland, which share a similar history of conversion, soil disturbances and erosion (Table 3). Nevertheless, to clearly understand and accurately identify the type of fungal communities associated with vegetation diversity and anthropogenic activities, application of DNA-based methods such as DNA amplicon sequencing would allow assigning functional guilds of the fungal communities and discerning the main player for the fungal OTU diversity in response to the vegetation type.

## Conclusion

We conclude that land use change significantly affects soil microbial diversity and community composition in the Ethiopian highland through its effects on soil biophysical and chemical attributes. The study has also revealed that the current unsystematic land management activities, e.g., the traditional agricultural practices, promote deterioration of soil quality and development of desertification. On the other hand, restoration activities, like area exclosure, could reinforce the rehabilitation of the ecosystem. The complex interaction of the soil microbial communities with biotic and abiotic factors in the tropical soil ecosystems warrants detailed investigation on the characterization of litter quality, aboveground biomass, and other soil quality parameters, and their correlation with soil microbial communities’ richness and functional diversity.

Molecular fingerprinting techniques offer an opportunity to measure the land use change effect on soil microbial community structure, especially when dealing with large number of samples. In this study, the ARISA method was applied to describe the soil microbial communities’ composition across different land use types. ARISA is a proven fingerprinting technique, which is useful for time-efficient sample processing and comparative analysis of microbial community structure. However, it has some limitations in terms of accurately describing microbial diversity and understanding the microbial functional diversity. This method is likely to underestimate species richness with increasing microbial communities’ diversity. Further studies in the Ethiopian highlands should consider the application of next generation sequencing (NGS) technology. On one hand, this will help to determine how the land use change drives the shift in soil microbial diversity and community composition and on another hand, its implication on the functional diversity of soil microbiomes in relation to spatial and temporal patterns of belowground processes. Such knowledge will be crucial in understanding the impact of land use changes, developing alternative land management mechanisms in order to maintain a healthy ecosystem or restore a degraded ecosystem.

## Additional file

**Additional file 1: Figure S1.** Land use-averaged rarefaction curves for bacteria and fungi. For each land use, rarefaction curves were generated using the seven replicates.

## Abbreviations

AMF-SD: arbuscular mycorrhizal fungi—spore density
ANOSIM: analysis of similarity
ARARI: Amhara Regional Agricultural Research Institute
ARISA: automated ribosomal intergenic spacer analysis
BC.PI: bicarbonate-reactive Phosphorous
BOKU: Universität für Bodenkultur Wien (University of Natural Resources and Life Sciences)
DNA: deoxyribonucleic acid
FAM: fluorescein amidite
IFE: Institute of Forest Ecology
INVAM: International Culture Collection of Arbuscular Mycorrhizal Fungi
NaHCO3: sodium bicarbonate
NaOH: sodium hydroxide
NMDS: non-metric multidimensional scaling
OC: organic carbon
OH.PI: hydroxide-reactive Phosphorous
OTUs: operational taxonomic units
PCR: polymerase chain reaction
RFI: relative fluorescent intensity
SAS: soil aggregate stability
SD: arbuscular mycorrhizal fungal spore density
TSN: total soil nitrogen
UFZ: Helmholtz-Zentrums für Umweltforschung (Helmholtz Centre for Environmental Research)
