# Supplementary material for: Transition of Ethiopian highland forests to agriculture-dominated landscapes shifts the soil microbial community composition
Source: BMC Ecol. 2018 Dec 17;18:58. doi: 10.1186/s12898-018-0214-8 (PMC6298011; doi:10.1186/s12898-018-0214-8)

**Additional file**

**Title:** Transition of Ethiopian highland forests to agriculture-dominated landscapes shifts the soil microbial community composition

Yoseph T. Delelegn^1^*^¶^, Witoon Purahong^2^*, Hans Sandén^1^, Birru Yitaferu^3^, Douglas L. Godbold^1^, Tesfaye Wubet^2, 4, †^

**Affiliations:**

^1^BOKU – University of Natural Resources and Life Sciences, Institute of Forest Ecology (IFE), Peter-Jordan-Straße 82, 1190 Vienna, Austria;

^2^UFZ-Helmholtz Centre for Environmental Research, Department of Soil Ecology, Theodor-Lieser-Str. 4, D-06120 Halle (Saale), Germany;

^3^ARARI–Amhara Regional Agricultural Research Institute, P. O. B. 527, Bahir Dar, Ethiopia;

^4^German Centre for Integrative Biodiversity Research (iDiv), Halle-Jena-Leipzig, Leipzig, Germany;

^†^Current address: Department of Community Ecology, UFZ-Helmholtz-Centre for Environmental Research, Halle/Saale, Germany.

E-mail addresses:

Yoseph T. Delelegn ([yosephjet@gmail.com](mailto:yosephjet@gmail.com)), Witoon Purahong ([witoon.purahong@ufz.de](mailto:witoon.purahong@ufz.de)), Hans Sandén ([hans.sanden@boku.ac.at](mailto:hans.sanden@boku.ac.at)), Birru Yitaferu ([birru_yitaferu2002@yahoo.com](mailto:birru_yitaferu2002@yahoo.com)), Douglas L. Godbold ([douglas.godbold@boku.ac.at](mailto:douglas.godbold@boku.ac.at)), Tesfaye Wubet ([tesfaye.wubet@ufz.de](mailto:tesfaye.wubet@ufz.de))

^¶^Correspondence and requests for materials should be addressed to Yoseph T. Delelegn (yoseph.delelegn@boku.ac.at / [yosephjet@gmail.com](mailto:yosephjet@gmail.com))

*Equal contribution

**Figure S1.** Land use-averaged rarefaction curves for bacteria and fungi. For each land use, rarefaction curves were generated using the seven replicates.


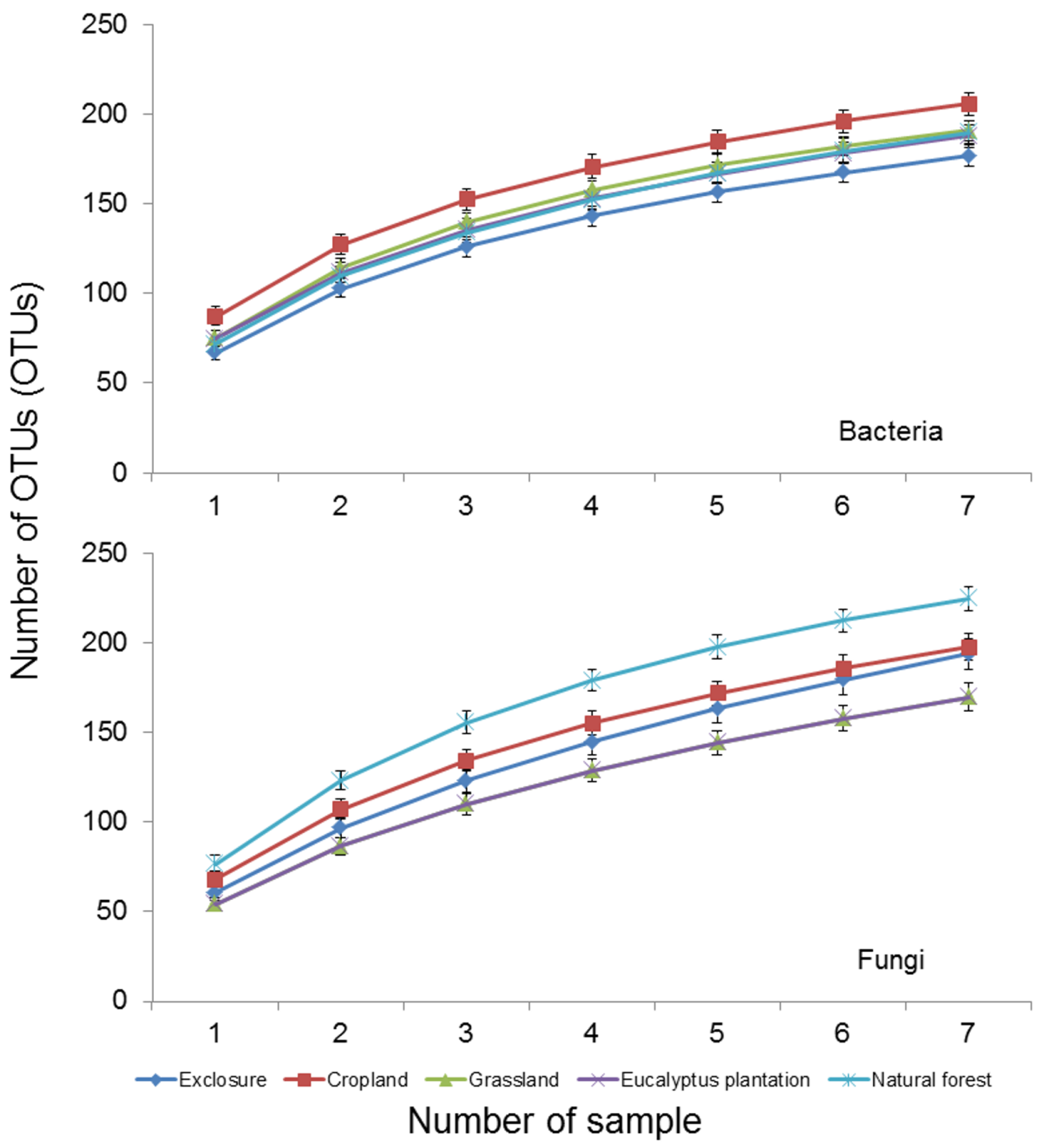

Supplement: Supplementary file 1 — Additional file 1: Figure S1. Land use-averaged rarefaction curves for bacteria and fungi. For each land use, rarefaction curves were generated using the seven replicates. [file 12898_2018_214_MOESM1_ESM.docx]
